# A Cell-Level Systems PK-PD Model to Characterize In Vivo Efficacy of ADCs

**DOI:** 10.3390/pharmaceutics11020098

**Published:** 2019-02-25

**Authors:** Aman P. Singh, Leiming Guo, Ashwni Verma, Gloria Gao-Li Wong, Dhaval K. Shah

**Affiliations:** 1 Department of Pharmaceutical Sciences, School of Pharmacy and Pharmaceutical Sciences, The State University of New York at Buffalo, 455 Kapoor Hall, Buffalo, New York, NY 14214-8033, USA; amanpree@buffalo.edu (A.P.S.); leimingg@buffalo.edu (L.G.); ashwnive@buffalo.edu (A.V.); 2 Department of Biological Sciences, The State University of New York at Buffalo, Buffalo, New York, NY 14214-8033, USA; gloriawo@buffalo.edu

**Keywords:** Antibody-drug conjugates, cellular pharmacokinetics, tumor pharmacokinetics, PK-PD model, in vivo efficacy, Trastuzumab-vc-MMAE, tubulin occupancy

## Abstract

Here, we have presented the development of a systems pharmacokinetics-pharmacodynamics (PK-PD) model for antibody-drug conjugates (ADCs), which uses intracellular target occupancy to drive in-vivo efficacy. The model is built based on PK and efficacy data generated using Trastuzumab-Valine-Citrulline-Monomethyl Auristatin E (T-vc-MMAE) ADC in N87 (high-HER2) and GFP-MCF7 (low-HER2) tumor bearing mice. It was observed that plasma PK of all ADC analytes was similar between the two tumor models; however, total trastuzumab, unconjugated MMAE, and total MMAE exposures were >10-fold, ~1.6-fold, and ~1.8-fold higher in N87 tumors. In addition, a prolonged retention of MMAE was observed within the tumors of both the mouse models, suggesting intracellular binding of MMAE to tubulin. A systems PK model, developed by integrating single-cell PK model with tumor distribution model, was able to capture all in vivo PK data reasonably well. Intracellular occupancy of tubulin predicted by the PK model was used to drive the efficacy of ADC using a novel PK-PD model. It was found that the same set of PD parameters was able to capture MMAE induced killing of GFP-MCF7 and N87 cells in vivo. These observations highlight the benefit of adopting a systems approach for ADC and provide a robust and predictive framework for successful clinical translation of ADCs.

## 1. Introduction

An ability to deliver potent chemotherapeutic agents to antigen expressing tumors via targeted monoclonal antibodies (mAbs) make antibody-drug conjugates (ADC) an attractive platform for anticancer drug development [1]. These molecules present an opportunity to expand the therapeutic index of chemotherapeutic agents, by enhancing their exposure at the site-of-action in the tumor and reducing the exposure inside toxicity prone normal tissues. With the advent of novel linker technologies [2], site-specific conjugation [3], and design of synthetic payloads (e.g., PBDs) [4], the clinical landscape of ADC is exponentially increasing with four drugs approved by the FDA and more than 80 molecules in clinical development [5]. While ADCs are very promising, their clinical success is not always guaranteed, and often challenged by lack of efficacy and severe dose-limiting toxicities [6]. Consequently, development of robust quantitative methods, such as pharmacokinetics-pharmacodynamics (PK-PD) modeling and simulation (M&S), can be very valuable for guiding a cost and time-effective development of ADCs.

There are several PK-PD models reported in the literature for ADCs [7,8], whose ability to predict novel scenarios and facilitate preclinical-to-clinical translation varies. In general, multiscale systems PK-PD models are preferred over empirical models [9,10], as they can incorporate key elements of the underlying system, which ultimately imparts quantitative and mechanistic rigor to these models. An ability to accurately characterize cellular disposition of ADCs is an essential component of these models, as this process is integral for successful delivery of payload into the tumor cells and subsequent cytotoxic effects. In fact, using multiple ADC molecules we have demonstrated that it is important to accurately characterize cellular disposition of ADCs for successfully predicting tumor exposures and clinical outcomes of ADCs using PK-PD M&S [11,12,13].

We have recently developed a single cell PK model for ADCs using Trastuzumab-Valine-Citrulline-Monomethyl Auristatin E (T-vc-MMAE) as a tool for ADC [14]. The model is mathematically unique in a sense that it could predict the catabolic fate of an ADC within a single cancer cell, among a growing population of tumor cells. Using known system parameters, such as target antigen expression, ADC binding affinity, internalization rate and intracellular degradation rate, the model was able to effectively characterize the relationship between extracellular ADC concentrations and intracellular exposure of different ADC analytes, in high and low HER2 expressing cells (i.e., HER2-high N87 and HER2-low GFP-MCF7 cells). The model was also expanded to include two different populations of cells in a coculture system, to characterize the bystander effect of ADCs. A unique in-vitro PK-PD relationship was developed using this model, which utilized intracellular occupancy of pharmacological target (tubulin) by the payload (MMAE) to characterize direct and bystander killing of cancer cells in a coculture system [15,16].

In this paper, we have extended the application of the single cell ADC PK-PD model towards in-vivo system. The in vivo PK model was developed by integrating the single cell PK model with our previously published tumor disposition model for ADCs [11,12,13]. Tumor disposition model was further integrated with a cell-level PK-PD model to develop an in-vivo systems PK-PD framework, where occupancy to tubulin by MMAE was used to drive the killing of tumor cells. The developed model was validated with the help of experimental data. In vivo PK studies were conducted using T-vc-MMAE ADC in GFP-MCF7 and N87 xenograft mouse models to obtain plasma and tumor PK of different ADC analytes (i.e., total trastuzumab, free MMAE, and total MMAE). In addition, tumor growth inhibition (TGI) studies were conducted in both xenograft models using different dose-levels of T-vc-MMAE to establish dose-response relationship for the ADC. All the experimental data was mathematically integrated using the proposed in-vivo systems PK-PD model for ADCs.

## 2. Materials and Methods

### 2.1. Cell Lines

The two HER2-expressing cell lines utilized for the development of tumor bearing mouse models were NCI-N87 cells (human gastric carcinoma cells) and green fluorescent protein (GFP) transfected MCF7 cells (breast cancer cells). HER2 expression for N87 and GFP-MCF7 cells has been reported to be around 950,000 and 55,000 receptors per cell, respectively [14], which makes them appropriate model systems for investigating high and low HER2 expressing tumors.

### 2.2. Tool ADC

The tool ADC utilized for current investigation was Trastuzumab-vc-MMAE, which was synthesized and characterized in-house. Commercially available Trastuzumab (Herceptin^®^, Genentech, South San Francisco, CA, USA) was conjugated with valine-citrulline MMAE (drug-linker solution) using random conjugation method, resulting in a heterogeneous formulation with an average drug: antibody ratio (DAR) of ~4. Detailed protocol for synthesis and characterization of T-vc-MMAE has been published before [14,16].

### 2.3. Development of Xenograft Mouse Models

Male severe combined immunodeficient (NOD.CB17-*Prkdc^scid^/J*) mice were purchased at the age of six weeks from Jackson Laboratory, ME, USA. After acclimation to their new housing conditions for two weeks, mice were subcutaneously implanted in the right dorsal flank with ~10 million tumor cells (N87 or GFP-MCF7) suspended in the growth medium with no FBS. To facilitate faster growth of GFP-MCF7 tumors, mice were supplemented with 1µg of 17β-estradiol valerate (Sigma^®^, St. Louis, MO, USA) in 50 µL of pharmaceutical grade peanut oil (Sigma^®^), by subcutaneous injection every 4 days [17] until the termination of the study. All procedures involving animals, including housing and care, method of euthanasia, and experimental protocols were conducted in accordance with the Institutional Animal Care and Use Committee (IACUC) at State University of New York at Buffalo. The permission for the animal experiments was provided by IACUC under the approval number PHC26114Y on 1 October 2014. 

### 2.4. Development of Bioanalytical Techniques

To characterize the plasma and tumor PK of T-vc-MMAE, three different bioanalytical methods were developed to measure total trastuzumab, total MMAE (conjugated and unconjugated), and free MMAE (unconjugated) in plasma and tumor matrices. A sandwich ELISA method was used to measure total intact trastuzumab levels, whereas LC-MS/MS based method was used to measure free (unconjugated) MMAE in plasma and tumor. A forced deconjugation method was utilized to release antibody-conjugated MMAE in a biological sample, which was then analyzed using the LC-MS/MS method to determine total MMAE in plasma and tumor samples. For bioanalysis, weighed tumor samples were homogenized in DI water with 1% protease inhibitor cocktail using a tissue homogenizer to a final concentration of 300 mg/mL. A typical blood sample was collected in tubes precoated with K_2_EDTA at 4 °C followed by centrifugation for 15 min at 1000 RPM. Plasma and tumor lysate sample were then divided into three groups associated with detection of each analyte. Standard curves were generated using plasma and tumor lysate samples from untreated animals and spiking with different concentrations of either trastuzumab (for ELISA) or MMAE (for LC-MS/MS). Detailed methodology, development, and validation of all three bioanalytical techniques have been published before [14].

### 2.5. Tumor Pharmacokinetic Studies

A total of 18 SCID mice were equally divided into two groups, which were inoculated with either GFP-MCF7 or N87 cells. Treatment was initiated four to six weeks after tumor implantation, when the tumors were ~500 mm^3^. All the animals from each group were treated with single 10 mg/kg dose of T-vc-MMAE intravenously. At 24 h, 72 h, and 168 h after ADC administration, three animals from each group were sacrificed to collect blood and tumor samples. In addition, a blood sample was also collected from each animal at 10 min after ADC dose via retro-orbital sampling. Blood samples were immediately processed to extract plasma, and all the plasma and tumor samples were stored at −80 °C until further analysis.

### 2.6. Tumor Growth Inhibition Studies

A total of 56 SCID mice were equally divided into two groups, which were inoculated with either GFP-MCF7 (Group A) or N87 (Group B) cells. Treatment with different dose-levels of T-vc-MMAE was initiated once the tumors reached ~350−400 mm^3^. On day 0, mice from each group (A and B) were further randomly divided into four equal subgroups (*n* = 7), either control (A1 and B1) or treatment (A2-4 and B2-4). GFP-MCF7 bearing mice were injected with a single intravenous dose of 3 mg/kg (A2), 5 mg/kg (A3) or 10 mg/kg (A4) ADC. N87 bearing mice were injected with a single intravenous dose of 1 mg/kg (B2), 3 mg/kg (B3), or 10 mg/kg (B4) ADC. Tumor volumes were calculated based on tumor length (L) and breadth (B) using the following formula: $\frac{1}{2}\times L\times B^{2}$. Tumor volumes in all the groups were measured twice a week until either tumor volume exceeded the permissible limit or was completely regressed for a prolonged duration of time (i.e., ~3 week).

### 2.7. Development of In-Vivo systems PK-PD Model for T-vc-MMAE ADC

#### 2.7.1. Plasma PK Model for ADC

**Figure 1:**
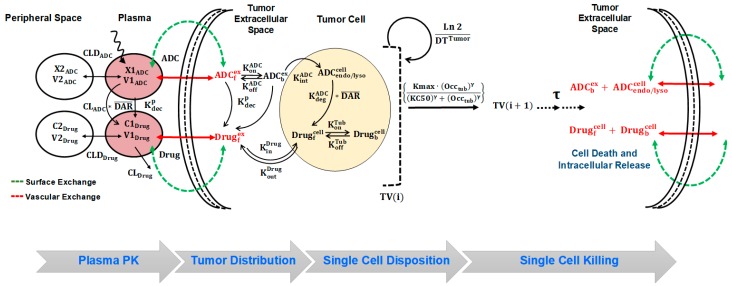
A schematic diagram of the systems pharmacokinetics-pharmacodynamics (PK-PD) model for antibody-drug conjugates (ADCs). ***Plasma PK:*** Disposition of Trastuzumab-Valine-Citrulline-Monomethyl Auristatin E (T-vc-MMAE) in systemic and peripheral spaces is characterized using a two-compartment model with linear clearance from the central compartment. Processes associated with non-specific shedding of MMAE and catabolic clearance of T-vc-MMAE contribute to the formation of unconjugated MMAE, which is also characterized using a two-compartment model with distribution to peripheral tissues and linear clearance from the central compartment. ***Tumor Distribution:*** the distribution of T-vc-MMAE and unconjugated MMAE was assumed to be driven from their central compartment to tumor extracellular space using two diffusive processes, i.e., surface and vascular exchange. ***Single Cell Disposition:*** once in the extracellular space, T-vc-MMAE was assumed to bind to HER2 receptors and internalize into the endosomal/lysosomal space of each cell. Upon enzymatic degradation and linker cleavage, unconjugated MMAE was assumed to release in the cytoplasmic space and either bind to intracellular tubulin or efflux out in the extracellular space. ***Single Cell Killing:*** occupancy of intracellular tubulin with MMAE drives the killing of cells and shuttles the growing cells into non-growing phases. Upon the death of each cell, the intracellular content becomes part of tumor extracellular space, which can distribute back into other cells or diffuse out in the systemic circulation.

Systemic PK of T-vc-MMAE was characterized using our previously published plasma PK model [10,11,12], a schematic that is described in Figure 1 and Figure S1. The biexponential profile of total trastuzumab and T-vc-MMAE is characterized using a 2-compartment model with linear catabolic clearance (${\mathrm{CL}}_{\mathrm{ADC}}$) from the central compartment. The distribution of T-vc-MMAE to peripheral tissues is characterized using a distributional clearance parameter, ${\mathrm{CLD}}_{\mathrm{ADC}}$. An additional first order elimination rate constant ($K_{\mathrm{dec}}^{P}$), coined as ‘non-specific deconjugation rate’, is incorporated in the central compartment to characterize non-specific deconjugation of MMAE from the ADC in the systemic circulation. This rate also governs first order decline in average DAR value over the time. Both elimination and deconjugation pathways (${\mathrm{CL}}_{\mathrm{ADC}}$ and $K_{\mathrm{dec}}^{P}$) releases free (unconjugated) MMAE, and hence, becomes the formation pathways for MMAE. The disposition of free MMAE is also characterized using a two-compartment model, which is parameterized with linear systemic clearance (${\mathrm{CL}}_{\mathrm{Drug}}$) and distributional clearance (${\mathrm{CLD}}_{\mathrm{Drug}}$).

#### 2.7.2. Tumor Distribution Model for ADC

Distribution of T-vc-MMAE and released MMAE in solid tumor is characterized using a tumor disposition model, of which the schematic is described in Figure 1 and Figure S2. Two distinct exchange processes (i.e., surface and vascular exchange) were incorporated to describe the mechanism of T-vc-MMAE and free MMAE distribution from systemic circulation to tumor extracellular space. Due to high interstitial pressure and lack of functional lymphatic system within the tumor microenvironment, it was assumed that the disposition of ADC and released drug in the tumor was limited to diffusive processes. Diffusion across the tumor surface was termed as the surface exchange and permeability across the tumor vasculature was termed as vascular exchange. It was assumed that size of the tumor determined the rate and pathway of ADC/released drug exchange with the tumor, where surface exchange predominates for smaller tumors and vascular exchange was more prominent for larger tumors. Since our PK-PD model also accounted for ADC-induced tumor regression, the relative contribution of these pathways towards sustaining ADC and released drug exposure within extracellular space varied with time. The diffusivity and permeability parameters for ADC and released drug were calculated using molecular size [18,19,20]. In addition, since the ADC/released drug only distribute to certain fraction of the whole tumor, the effective higher concentrations of ADC/released drug in the tumor were calculated using the “void volume (ε)” parameter, which is unique for the ADC and the released drug [11,12,13].

#### 2.7.3. Single Cell Disposition Model for ADC

Once in the tumor extracellular space, the disposition of T-vc-MMAE and released MMAE within each tumor cell is characterized using our single-cell disposition model for ADCs [14]. The key mechanistic components incorporated within the model includes T-vc-MMAE binding kinetics ($K_{\mathrm{on}}^{\mathrm{ADC}}$ and $K_{\mathrm{off}}^{\mathrm{ADC}}$) to HER2 receptors on tumor cell, followed by internalization ($K_{\mathrm{int}}^{\mathrm{ADC}}$) and intracellular degradation ($K_{\deg}^{\mathrm{ADC}}$) in the endosomal/lysosomal space, leading to the release of free (unconjugated) MMAE in the cytoplasm. The released MMAE within the cytoplasmic space is assumed to either bind to intracellular tubulin ($K_{\mathrm{on}}^{\mathrm{Tub}}$ and $K_{\mathrm{off}}^{\mathrm{Tub}}$) or exchange with the extracellular space using influx ($K_{\mathrm{in}}^{\mathrm{Drug}}$) and efflux ($K_{\mathrm{out}}^{\mathrm{Drug}}$) processes. To conserve the mass-balance within the dynamic system of growing tumor cells, a dilution factor is incorporated within the single cell equations which renders dilution of intracellular content (either intact ADC or released drug) at the rate equivalent to the growth rate ($K_{g}^{\mathrm{Tumor}}$) of each tumor-type. With an assumption of ~10^8^ tumor cells per gram of tumor [21], simultaneous interaction of T-vc-MMAE and free MMAE in the extracellular and intracellular space of each tumor cell is accounted for. The equations pertaining to the growth of the tumor (TV, in L) in the absence of any tumor growth inhibition are provided below (Equations (1) and (2)):

(1) $$\frac{d\left({\mathrm{TV}}_{\mathrm{mm}3}\right)}{\mathrm{dt}}=\frac{0.693}{{\mathrm{DT}}^{\mathrm{tumor}}}\cdot {\mathrm{TV}}_{\mathrm{mm}3}\;\;\;\mathrm{IC}={\mathrm{TV}}_{\mathrm{mm}3}\;\left(0\right)$$

(2) $${\mathrm{TV}}_{\mathrm{mm}3}=\mathrm{TV}\cdot 10^{6}\;\;\;R_{\mathrm{Tumor}}={\left[\frac{3\cdot \mathrm{TV}\cdot 1000}{4\pi }\right]}^{1/3}$$

Equations associated with plasma PK and tumor distribution of T-vc-MMAE (in amounts) and unconjugated MMAE (in concentrations) are provided below (Equations (3–7)):

(3) $$\begin{array}{l} \frac{d\left(X1_{\mathrm{ADC}}\right)}{\mathrm{dt}}=-\frac{{\mathrm{CL}}_{\mathrm{ADC}}}{V1_{\mathrm{ADC}}}\;\cdot X1_{\mathrm{ADC}}-\frac{{\mathrm{CLD}}_{\mathrm{ADC}}}{V1_{\mathrm{ADC}}}\cdot X1_{\mathrm{ADC}}+\frac{{\mathrm{CLD}}_{\mathrm{ADC}}}{V2_{\mathrm{ADC}}}\cdot X2_{\mathrm{ADC}}-K_{\mathrm{dec}}^{P}\cdot X1_{\mathrm{ADC}}\\ \;\;\;\;\;-\left(\frac{X1_{\mathrm{ADC}}}{V1_{\mathrm{ADC}}}-\frac{{\mathrm{ADC}}_{f}^{\mathrm{ex}}}{\varepsilon^{\mathrm{ADC}}}\right)\cdot \mathrm{TV}\cdot \left(\frac{2\cdot P_{\mathrm{ADC}}\cdot R_{\mathrm{Cap}}}{R_{\mathrm{Krogh}}^{2}}+\frac{6\cdot D_{\mathrm{ADC}}}{R_{\mathrm{Tumor}}^{2}}\right) \end{array}$$

(4) $$\frac{d\left(X2_{\mathrm{ADC}}\right)}{\mathrm{dt}}=\frac{{\mathrm{CLD}}_{\mathrm{ADC}}}{V1_{\mathrm{ADC}}}\cdot X1_{\mathrm{ADC}}-\frac{{\mathrm{CLD}}_{\mathrm{ADC}}}{V2_{\mathrm{ADC}}}\cdot X2_{\mathrm{ADC}}$$

(5) $$\begin{array}{ll} \frac{d\left(C1_{\mathrm{Drug}}\right)}{\mathrm{dt}}=- & \frac{{\mathrm{CL}}_{\mathrm{Drug}}}{V1_{\mathrm{Drug}}}\cdot C1_{\mathrm{Drug}}-\frac{{\mathrm{CLD}}_{\mathrm{Drug}}}{V1_{\mathrm{Drug}}}\cdot C1_{\mathrm{Drug}}+\frac{{\mathrm{CLD}}_{\mathrm{Drug}}}{V1_{\mathrm{Drug}}}\cdot C2_{\mathrm{Drug}}+\frac{\left(K_{\mathrm{dec}}^{P}\cdot X1_{\mathrm{ADC}}\cdot \bar{\mathrm{DAR}}\right)}{V1_{\mathrm{Drug}}}\\ & +\frac{{\mathrm{CL}}_{\mathrm{ADC}}\cdot \bar{\mathrm{DAR}}\cdot \frac{X1_{\mathrm{ADC}}}{V1_{\mathrm{ADC}}}}{V1_{\mathrm{Drug}}}-\left(C1_{\mathrm{Drug}}-\frac{{\mathrm{Drug}}_{f}^{\mathrm{ex}}}{\left(\mathrm{TV}\cdot \varepsilon^{\mathrm{Drug}}\right)}\right)\\ & \cdot (\frac{2\cdot P_{\mathrm{Drug}}\cdot R_{\mathrm{Cap}}}{R_{\mathrm{Krogh}}^{2}}+\frac{6\cdot D_{\mathrm{Drug}}}{R_{\mathrm{Tumor}}^{2}}) \end{array}$$

(6) $$\frac{d\left(C2_{\mathrm{Drug}}\right)}{\mathrm{dt}}=\frac{{\mathrm{CLD}}_{\mathrm{Drug}}}{V2_{\mathrm{Drug}}}\cdot C1_{\mathrm{Drug}}-\frac{{\mathrm{CLD}}_{\mathrm{Drug}}}{V2_{\mathrm{Drug}}}\cdot C2_{\mathrm{Drug}}$$

(7) $$\frac{d\left(\bar{\mathrm{DAR}}\right)}{\mathrm{dt}}=-K_{\mathrm{dec}}^{P}\cdot \bar{\mathrm{DAR}}$$

The initial conditions for Equations (3) and (7) are ${\mathrm{Dose}}^{\mathrm{ADC}}$ (injected intravenous dose of ADC) and $\bar{\mathrm{DAR}}\left(0\right)$ (initial average DAR value of the ADC in the formulation), respectively. The initial conditions for the rest of equations are zero. Equations associated with the concentration of T-vc-MMAE and amounts of unconjugated MMAE in the tumor extracellular space are listed below (Equations (8) and (9)):

(8) $$\begin{array}{l} \frac{d\left({\mathrm{ADC}}_{f}^{\mathrm{ex}}\right)}{\mathrm{dt}}=\left(\frac{X1_{\mathrm{ADC}}}{V1_{\mathrm{ADC}}}-\frac{{\mathrm{ADC}}_{f}^{\mathrm{ex}}}{\varepsilon^{\mathrm{ADC}}}\right)\cdot \left(\frac{2\cdot P_{\mathrm{ADC}}\cdot R_{\mathrm{Cap}}}{R_{\mathrm{Krogh}}^{2}}+\frac{6\cdot D_{\mathrm{ADC}}}{R_{\mathrm{Tumor}}^{2}}\right)\\ \;\;\;\;\;\;\;\;+\left(-K_{\mathrm{on}}^{\mathrm{ADC}}\cdot \frac{{\mathrm{ADC}}_{f}^{\mathrm{ex}}}{\varepsilon^{\mathrm{ADC}}}\cdot \left({\mathrm{Ag}}^{\mathrm{Ex}}-{\mathrm{ADC}}_{b}^{\mathrm{Cell}}\right)+K_{\mathrm{off}}^{\mathrm{ADC}}\cdot {\mathrm{ADC}}_{b}^{\mathrm{Cell}}\right)\cdot \frac{{\mathrm{NC}}^{\mathrm{Tumor}}\cdot \mathrm{SF}}{\mathrm{TV}}\\ \;\;\;\;\;\;\;\;-K_{\mathrm{dec}}^{p}\cdot {\mathrm{ADC}}_{f}^{\mathrm{ex}} \end{array}$$

(9) $$\begin{array}{l} \frac{d\left({\mathrm{Drug}}_{f}^{\mathrm{ex}}\right)}{\mathrm{dt}}=\left(C1_{\mathrm{Drug}}-\frac{{\mathrm{Drug}}_{f}^{\mathrm{ex}}}{\left(\mathrm{TV}\cdot \varepsilon^{\mathrm{Drug}}\right)}\right)\cdot \mathrm{TV}\cdot \left(\frac{2\cdot P_{\mathrm{Drug}}\cdot R_{\mathrm{Cap}}}{R_{\mathrm{Krogh}}^{2}}+\frac{6\cdot D_{\mathrm{Drug}}}{R_{\mathrm{Tumor}}^{2}}\right)+K_{\mathrm{dec}}^{p}\cdot {\mathrm{ADC}}_{f}^{\mathrm{ex}}\\ \;\;\;\;\;\;\;\;\cdot \bar{\mathrm{DAR}}\cdot \mathrm{TV}+\left(K_{\mathrm{dec}}^{p}\cdot {\mathrm{ADC}}_{b}^{\mathrm{Cell}}\cdot \bar{\mathrm{DAR}}+K_{\mathrm{out}}^{\mathrm{Drug}}\cdot {\mathrm{Drug}}_{f}^{\mathrm{Cell}}\right)\cdot {\mathrm{NC}}^{\mathrm{Tumor}}\cdot \mathrm{SF}\\ \;\;\;\;\;\;\;\;-K_{\mathrm{in}}^{\mathrm{Drug}}\cdot {\mathrm{NC}}^{\mathrm{Tumor}}\cdot \left(\frac{V^{\mathrm{Cell}}}{\mathrm{TV}\cdot \varepsilon^{\mathrm{Drug}}}\right)\cdot {\mathrm{Drug}}_{f}^{\mathrm{ex}} \end{array}$$

Equations associated with the cellular disposition of T-vc-MMAE and released MMAE in the form of the number of molecules of each ADC species in a single tumor cell, are provided below (Equations (10–13)):

(10) $$\begin{array}{l} \frac{d\left({\mathrm{ADC}}_{b}^{\mathrm{Cell}}\right)}{\mathrm{dt}}=K_{\mathrm{on}}^{\mathrm{ADC}}\cdot \frac{{\mathrm{ADC}}_{f}^{\mathrm{ex}}}{\varepsilon^{\mathrm{ADC}}}\cdot \left({\mathrm{Ag}}^{\mathrm{Ex}}-{\mathrm{ADC}}_{b}^{\mathrm{Cell}}\right)-K_{\mathrm{off}}^{\mathrm{ADC}}\cdot {\;\mathrm{ADC}}_{b}^{\mathrm{Cell}}-\left(K_{\mathrm{dec}}^{p}+K_{\mathrm{int}}^{\mathrm{ADC}}\right)\cdot {\mathrm{ADC}}_{b}^{\mathrm{Cell}}\\ \;\;\;\;\;\;-\frac{0.693}{{\mathrm{DT}}^{\mathrm{Tumor}}}\cdot {\mathrm{ADC}}_{b}^{\mathrm{Cell}} \end{array}$$

(11) $$\frac{d\left({\mathrm{ADC}}_{\mathrm{lyso}}^{\mathrm{Cell}}\right)}{\mathrm{dt}}=K_{\mathrm{int}}^{\mathrm{ADC}}\cdot {\mathrm{ADC}}_{b}^{\mathrm{Cell}}-K_{\deg}^{\mathrm{ADC}}\cdot {\mathrm{ADC}}_{\mathrm{lyso}}^{\mathrm{Cell}}-\frac{0.693}{{\mathrm{DT}}^{\mathrm{Tumor}}}\cdot {\mathrm{ADC}}_{\mathrm{lyso}}^{\mathrm{Cell}}$$

(12) $$\begin{array}{ll} \frac{d\left({\mathrm{Drug}}_{f}^{\mathrm{Cell}}\right)}{\mathrm{dt}}= & K_{\deg}^{\mathrm{ADC}}\cdot {\mathrm{ADC}}_{\mathrm{lyso}}^{\mathrm{Cell}}\cdot \bar{\mathrm{DAR}}-K_{\mathrm{out}}^{\mathrm{Drug}}\cdot {\mathrm{Drug}}_{f}^{\mathrm{Cell}}-K_{\mathrm{on}}^{\mathrm{Tub}}\cdot {\mathrm{Drug}}_{f}^{\mathrm{Cell}}\cdot \left({\mathrm{Tub}}^{\mathrm{Tot}}-{\mathrm{Drug}}_{b}^{\mathrm{Cell}}\right)\\ & +K_{\mathrm{off}}^{\mathrm{Tub}}\cdot {\mathrm{Drug}}_{b}^{\mathrm{Cell}}+K_{\mathrm{in}}^{\mathrm{Drug}}\cdot \left(\frac{V^{\mathrm{Cell}}}{\mathrm{TV}\cdot \varepsilon^{\mathrm{Drug}}}\right)\cdot \left(\frac{{\mathrm{Drug}}_{f}^{\mathrm{ex}}}{\mathrm{SF}}\right)-\frac{0.693}{{\mathrm{DT}}^{\mathrm{Tumor}}}\cdot {\mathrm{Drug}}_{f}^{\mathrm{Cell}} \end{array}$$

(13) $$\frac{d\left({\mathrm{Drug}}_{b}^{\mathrm{Cell}}\right)}{\mathrm{dt}}=K_{\mathrm{on}}^{\mathrm{Tub}}\cdot {\mathrm{Drug}}_{f}^{\mathrm{Cell}}\cdot \left({\mathrm{Tub}}^{\mathrm{Tot}}-{\mathrm{Drug}}_{b}^{\mathrm{Cell}}\right)-K_{\mathrm{off}}^{\mathrm{Tub}}\cdot {\mathrm{Drug}}_{b}^{\mathrm{Cell}}-\frac{0.693}{{\mathrm{DT}}^{\mathrm{Tumor}}}\cdot {\mathrm{Drug}}_{b}^{\mathrm{Cell}}$$

The initial conditions for Equations (8–13) are zero.

#### 2.7.4. Characterization of Intracellular Occupancy of Tubulin with MMAE

Upon integration of the tumor distribution model with a single cell PK model for T-vc-MMAE, the percent occupancy of tubulin by MMAE inside each tumor cell can be calculated (Equation (14)) by dividing the number of tubulin-bound MMAE molecules with the number of total tubulin molecules inside each cell:

(14) $${\mathrm{Occ}}_{\mathrm{Tub}}=\left(\frac{{\mathrm{Drug}}_{b}^{\mathrm{Cell}}}{{\mathrm{Tub}}^{\mathrm{total}}}\right)\cdot 100$$

#### 2.7.5. Linking Intracellular Occupancy of Tubulin to Tumor-Growth Inhibition

TGI data obtained at different ADC dose levels were utilized to develop the in vivo systems PK-PD model shown in Figure 1. The integrated tumor distribution and cell-level PK model was used to derive intracellular occupancy of tubulin by MMAE molecules released in the cytoplasm. The intracellular occupancy was then utilized to drive the tumor growth inhibition using a non-linear killing function, which shuttles the growing tumor cells into non-growing phases, eventually leading to their death. This phenomenon is often depicted in the literature as ‘cell-distribution model’ [22]. It was also assumed that among all different cell-distribution populations (growing or non-growing) of cells, the cellular processing of ADC was active, and upon the death of tumor cells the intracellular content (either intact T-vc-MMAE or free MMAE) comes out and becomes part of the tumor extracellular space. The released intracellular content was allowed to re-distribute into other cancer cells or diffuse out of the tumor into the systemic circulation via surface or vascular exchange processes (Figure 1).

The resulting equations characterizing tumor growth and killing are provided below (Equations (15–21)):

Growth rate:

(15) $$\mathrm{Kg}=\left(\frac{0.693}{{\mathrm{DT}}^{\mathrm{tumor}}}\right)$$

Killing rate:

(16) $$\mathrm{Kill}=\left\{\frac{\mathrm{Kmax}\cdot {\left({\mathrm{Occ}}_{\mathrm{tub}}\right)}^{\gamma }}{{\left(\mathrm{KC}50\right)}^{\gamma }+{\left({\mathrm{Occ}}_{\mathrm{tub}}\right)}^{\gamma }}\right\}$$

(17) $$\frac{d\left({\mathrm{TV}}_{\mathrm{mm}3}^{1}\right)}{\mathrm{dt}}=\left(\mathrm{Kg}-\mathrm{Kill}\right)\cdot {\mathrm{TV}}_{\mathrm{mm}3}^{1}$$

(18) $$\frac{d\left({\mathrm{TV}}_{\mathrm{mm}3}^{2}\right)}{\mathrm{dt}}=\mathrm{Kill}\cdot {\mathrm{TV}}_{\mathrm{mm}3}^{1}-\frac{1}{\tau }\cdot {\mathrm{TV}}_{\mathrm{mm}3}^{2}$$

(19) $$\frac{d\left({\mathrm{TV}}_{\mathrm{mm}3}^{3}\right)}{\mathrm{dt}}=\frac{1}{\tau }\cdot \left({\mathrm{TV}}_{\mathrm{mm}3}^{2}-{\mathrm{TV}}_{\mathrm{mm}3}^{3}\right)$$

(20) $$\frac{d\left({\mathrm{TV}}_{\mathrm{mm}3}^{4}\right)}{\mathrm{dt}}=\frac{1}{\tau }\cdot \left({\mathrm{TV}}_{\mathrm{mm}3}^{3}-{\mathrm{TV}}_{\mathrm{mm}3}^{4}\right)$$

(21) $${\mathrm{TV}}_{\mathrm{mm}3}={\mathrm{TV}}_{\mathrm{mm}3}^{1}+{\mathrm{TV}}_{\mathrm{mm}3}^{2}+{\mathrm{TV}}_{\mathrm{mm}3}^{3}+{\mathrm{TV}}_{\mathrm{mm}3}^{4}$$

The equations for the concentration of T-vc-MMAE (Equation (8)) and amount of MMAE (Equation (9)) in tumor extracellular space were updated to account for the input of intracellular content from the dying cells in the last transit compartment (${\mathrm{TV}}_{\mathrm{mm}3}^{4}$). The resulting equations are provided below (Equations (22) and (23)):

(22) $$\begin{array}{l} \frac{d\left({\mathrm{ADC}}_{f}^{\mathrm{ex}}\right)}{\mathrm{dt}}=\left(\frac{X1_{\mathrm{ADC}}}{V1_{\mathrm{ADC}}}-\frac{{\mathrm{ADC}}_{f}^{\mathrm{ex}}}{\varepsilon^{\mathrm{ADC}}}\right)\cdot \left(\frac{2\cdot P_{\mathrm{ADC}}\cdot R_{\mathrm{Cap}}}{R_{\mathrm{Krogh}}^{2}}+\frac{6\cdot D_{\mathrm{ADC}}}{R_{\mathrm{Tumor}}^{2}}\right)\\ \;\;\;\;\;\;+\left(-K_{\mathrm{on}}^{\mathrm{ADC}}\cdot \frac{{\mathrm{ADC}}_{f}^{\mathrm{ex}}}{\varepsilon^{\mathrm{ADC}}}\cdot \left({\mathrm{Ag}}^{\mathrm{Ex}}-{\mathrm{ADC}}_{b}^{\mathrm{Cell}}\right)+K_{\mathrm{off}}^{\mathrm{ADC}}\cdot {\mathrm{ADC}}_{b}^{\mathrm{Cell}}\right)\cdot \frac{{\mathrm{NC}}^{\mathrm{Tumor}}\cdot \mathrm{SF}}{\mathrm{TV}}\\ \;\;\;\;\;\;-K_{\mathrm{dec}}^{p}\cdot {\mathrm{ADC}}_{f}^{\mathrm{ex}}+\frac{1}{\tau }\;\cdot {\mathrm{TV}}_{\mathrm{mm}3}^{4}\cdot 10^{5}\cdot \left({\mathrm{ADC}}_{b}^{\mathrm{Cell}}+{\mathrm{ADC}}_{\mathrm{lyso}}^{\mathrm{Cell}}\right)\cdot \frac{\mathrm{SF}}{\mathrm{TV}} \end{array}$$

(23) $$\begin{array}{l} \frac{d\left({\mathrm{Drug}}_{f}^{\mathrm{ex}}\right)}{\mathrm{dt}}=\left(C1_{\mathrm{Drug}}-\frac{{\mathrm{Drug}}_{f}^{\mathrm{ex}}}{\left(\mathrm{TV}\cdot \varepsilon^{\mathrm{Drug}}\right)}\right)\cdot \mathrm{TV}\cdot \left(\frac{2\cdot P_{\mathrm{Drug}}\cdot R_{\mathrm{Cap}}}{R_{\mathrm{Krogh}}^{2}}+\frac{6\cdot D_{\mathrm{Drug}}}{R_{\mathrm{Tumor}}^{2}}\right)+K_{\mathrm{dec}}^{P}\cdot {\mathrm{ADC}}_{f}^{\mathrm{ex}}\\ \;\;\;\;\;\;\cdot \bar{\mathrm{DAR}}\cdot \mathrm{TV}+\left(K_{\mathrm{dec}}^{p}\cdot {\mathrm{ADC}}_{b}^{\mathrm{Cell}}\cdot \bar{\mathrm{DAR}}+K_{\mathrm{out}}^{\mathrm{Drug}}\cdot {\mathrm{Drug}}_{f}^{\mathrm{Cell}}\right)\cdot {\mathrm{NC}}^{\mathrm{Tumor}}\cdot \mathrm{SF}\\ \;\;\;\;\;\;-K_{\mathrm{in}}^{\mathrm{Drug}}\cdot {\mathrm{NC}}^{\mathrm{Tumor}}\left(\frac{V^{\mathrm{Cell}}}{\mathrm{TV}\cdot \varepsilon^{\mathrm{Drug}}}\right)\cdot {\mathrm{Drug}}_{f}^{\mathrm{ex}}+\frac{1}{\tau }\cdot {\;\mathrm{TV}}_{\mathrm{mm}3}^{4}\cdot 10^{5}\cdot ({\mathrm{Drug}}_{f}^{\mathrm{Cell}}\\ \;\;\;\;\;\;+{\mathrm{Drug}}_{b}^{\mathrm{Cell}})\cdot \mathrm{SF} \end{array}$$

## 3. Parameter Estimation, Model Fitting and Simulations

The model development was conducted sequentially, where first plasma PK of T-vc-MMAE and unconjugated MMAE was used to estimate the parameters for the systemic PK model of ADC (Figure S1). Subsequently, the plasma PK parameters were fixed and the model was expanded to include the tumor distribution and cellular disposition of T-vc-MMAE (Figure S2). The tumor disposition model was validated using the tumor PK data, where only the parameter related to HER2 expression (${\mathrm{Ag}}^{\mathrm{ex}}$) in GFP-MCF7 and N87 tumors was estimated to account for in vivo evolution of the tumor. The validated tumor distribution model was integrated with the tumor growth inhibition (TGI) model, to facilitate the estimation of efficacy parameters (i.e., Kmax, KC50, τ and γ) for MMAE induced cell killing, which were kept the same for both the cell lines. An inter-individual variability (IIV) was estimated for the killing rate (Kmax) and transit-time (τ) parameters to account for the considerable variability observed between the animals during the TGI experiments.

Model development and simulation was performed using Berkeley Madonna^®^ (University of California at Berkeley, Berkeley, CA, USA), and PK data fitting was performed using maximum likelihood (ML) estimation method of ADAPT-5 software (BMSR, Los Angeles, CA, USA) [23]. The following variance model (Equation (24)) was used:

(24) $$\mathrm{Var}\left(t\right)={\left(\sigma_{\mathrm{intercept}}+\sigma_{\mathrm{slope}}\cdot Y\left(t\right)\right)}^{2}$$

The fitting of TGI data was obtained using the Stochastic Approximation Expectation Maximization (SAEM) algorithm of Monolix version 8 (Lixoft^®^) [24], where log-normal distribution was assumed for IIV in Kmax and τ parameters.

## 4. Results

### 4.1. Plasma and Tumor PK Studies:

**Figure 2:**
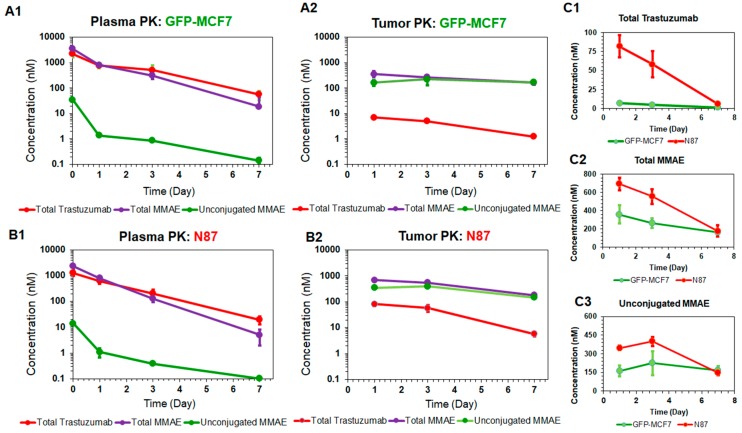
Plasma and tumor pharmacokinetic data (mean ± standard deviation) for three analytes (*n* = 3): unconjugated MMAE (green), total MMAE (purple), and total trastuzumab (red), after 10 mg/kg intravenous dose of T-vc-MMAE in GFP-MCF7 (**A1** and **A2**) and N87 (**B1** and **B2**) tumor bearing mice. Comparative tumor pharmacokinetics of 10 mg/kg T-vc-MMAE in GFP-MCF7 (green) and N87 (red) tumors for total trastuzumab (**C1**), total MMAE (**C2**) and unconjugated MMAE (**C3**).

Figure 2 depicts the PK profiles of total trastuzumab, unconjugated MMAE, and total MMAE, in the plasma and tumor homogenates of GFP-MCF7 (2A) and N87 (2B) xenografts. The plasma PK profiles of all three analytes were very similar between GFP-MCF7 and N87 xenografts (Figure 2A1 versus 2B1). Based on the non-compartmental analysis (NCA), total trastuzumab eliminated from the plasma with half-life of ~ 1.4 days in GFP-MCF7 bearing mice versus ~1.2 days in N87 bearing mice. The relatively higher clearance of antibody in SCID mice was attributed to the higher Fcγ mediated elimination of humanized IgG1 in these mice [25]. Total MMAE (unconjugated + mAb-conjugated) profiles in plasma eliminated with an approximate half-life of ~0.9 days in GFP-MCF7 bearing mice versus ~0.8 days in N87 bearing mice. The half-life of total MMAE was shorter than total trastuzumab due to the loss of MMAE from the ADC because of the non-specific deconjugation process. Unconjugated MMAE demonstrated a similar half-life of ~1.07 days versus ~1.14 days in GFP-MCF7 and N87 bearing mice. The longer half-life of MMAE is consistent with the formation-rate limited elimination of unconjugated MMAE.

In the tumor, the elimination half-life of total trastuzumab was ~2.14 days in GFP-MC7 bearing mice versus ~1.58 days in N87 bearing mice. There was a strong retention of MMAE observed in both the tumors when compared to the plasma. This retention mainly stems from intra-tumoral binding of MMAE, which helps MMAE sustain in the tumor for longer duration of time compared to the plasma. In fact, this observation is consistent with the findings from our previously published cellular disposition studies of T-vc-MMAE [14], where it was found that MMAE binds to intracellular tubulin and is retained within the cell for a prolonged period of time. When comparing the ratios of overall tumor exposure ($\mathrm{area}\;\mathrm{under}\;\mathrm{the}\;\mathrm{curve},{\;\mathrm{AUC}}_{0}^{7d}$) of all three analytes between high-HER2 and low-HER2 tumors (Figure 2C), it was observed that there was ~12-fold higher exposure of total trastuzumab, ~1.6-fold higher exposure of unconjugated MMAE, and ~1.8-fold higher exposure of total MMAE in N87 tumors compared to the GFP-MCF7 tumors. The diminished differential exposure for unconjugated and total MMAE in N87 tumors (compared to the total trastuzumab exposure) also accentuated the contribution of saturable intracellular binding of MMAE with tubulin.

### 4.2. Tumor Growth Inhibition Studies:

**Figure 3:**
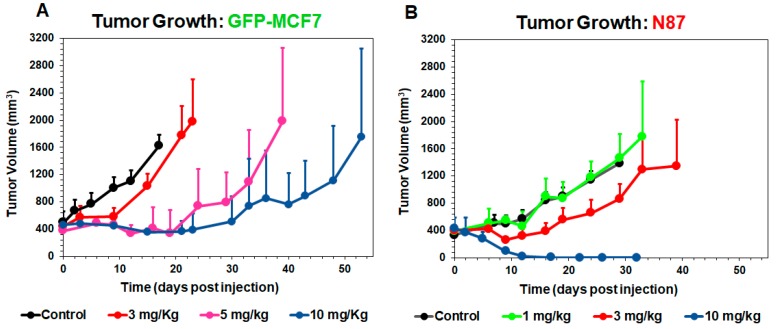
Tumor growth inhibition (TGI) profiles (mean ± standard deviation) in GFP-MCF7 (**A**) and N87 (**B**) tumors of either control (black, *n* = 7) or after single intravenous administration of T-vc-MMAE at 1 mg/kg (green, *n* = 7)), 3 mg/kg (red, *n* = 7), 5 mg/kg (pink, *n* = 7), and 10 mg/kg (blue, *n* = 7).

Figure 3 shows T-vc-MMAE induced tumor growth inhibition in GFP-MCF7 (Figure 3A) and N87 (Figure 3B) xenograft bearing mice, at doses ranging from 1–10 mg/kg. Overall, T-vc-MMAE was more efficacious in N87 (HER2-high) tumors compared to GFP-MCF7 (HER2-low) tumors. In GFP-MCF7 tumor bearing mice, the highest dose of 10 mg/kg was able to induce tumor stasis for a prolonged period. Time to achieve >1000 mm^3^ tumor volume for these mice was found to be 15, 33, and 48 days for 3, 5, and 10 mg/kg dose groups, compared to nine days for the control group. In N87 tumor bearing mice, there was an evident tumor regression at the higher dose-levels, and 10 mg/kg dose resulted in complete regression of the tumor. Time to achieve >1000 mm^3^ tumor volume for these mice was found to be 24 and 33 days for 1 and 3 mg/kg dose groups, compared to 24 days for the control group.

### 4.3. Development of the Systems PK-PD Model for ADC:

#### 4.3.1. Plasma PK Model for T-vc-MMAE:

**Figure 4:**
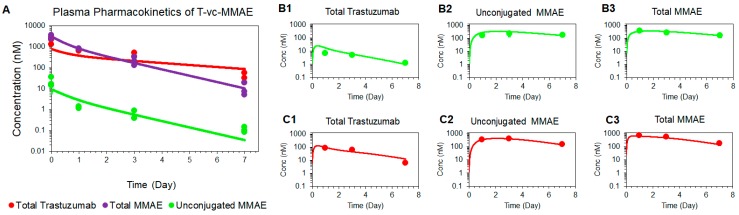
Model fittings for plasma and tumor pharmacokinetics of 10 mg/kg of intravenous 10 mg/kg T-vc-MMAE (**A**) Observed and model fitted profiles for plasma pharmacokinetics of total trastuzumab (red), total MMAE (purple), and unconjugated MMAE (green) in GFP-MCF7 and N87 tumor bearing mice. (**B** and **C**) Observed and model fitted profiles for tumor pharmacokinetics of total trastuzumab (**B1** and **C1**), free MMAE (**B2** and **C2**), and total MMAE (**B3** and **C3**) in GFP-MCF7 (green) and N87 (red) tumor bearing mice.

Figure 4A shows the observed and model fitted PK profiles of all three analytes of T-vc-MMAE in the plasma. The model was able to simultaneously characterize the PK of all three analytes reasonably well, using a pooled dataset from GFP-MCF7 and N87 tumor bearing mice. Parameters associated with the systemic disposition of unconjugated MMAE were fixed to previously reported values [11]. The rest of the parameters associated with the systemic disposition of T-vc-MMAE and non-specific deconjugation of MMAE ($K_{\mathrm{dec}}^{P}$) were estimated with good precision (Table 1).

#### 4.3.2. Tumor Distribution Model for T-vc-MMAE

Figure 4B,C shows the observed and model fitted PK profiles of all three analytes of T-vc-MMAE in GFP-MCF7 (Figure 4B) and N87 (Figure 4C) tumors. The model was able to capture the faster degrading profile of total trastuzumab (Figure 4(B1,C1)), and prolonged retention of unconjugated (Figure 4(B2,C2)) and total MMAE (Figure 4(B3,C3)) within the tumor reasonably well. Most of the parameters associated with tumor and cellular disposition of ADC were fixed a priori, and only the number of HER2 receptors on GFP-MCF7 and N87 tumor cells were estimated. These values were found to be 22,400 and 185,000 for GFP-MCF7 and N87 tumors, respectively (Table 1).

#### 4.3.3. Prediction of Intracellular Occupancy of Tubulin

**Figure 5:**
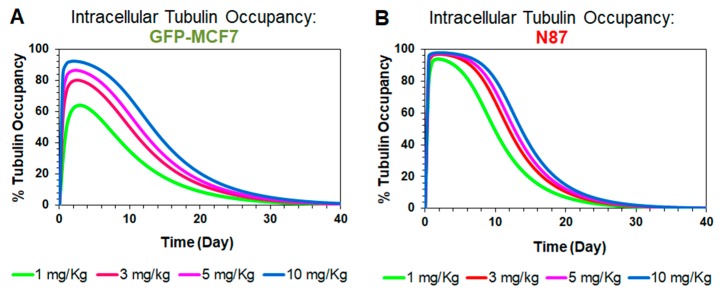
Simulations for intracellular occupancy of tubulin with MMAE (${\mathrm{Occ}}_{\mathrm{Tub}}$) in GFP-MCF7 (**A**) and N87 (**B**) tumor bearing mice after administration of single intravenous dose of 1 mg/kg (green), 3 mg/kg (red), 5 mg/kg (pink), and 10 mg/kg (blue) T-vc-MMAE.

Figure 5 shows model simulated profiles for intracellular occupancy of tubulin (${\mathrm{Occ}}_{\mathrm{Tub}}$) by MMAE. These profiles were generated using the tumor distribution model validated for GFP-MCF7 (Figure 5A) and N87 (Figure 5B) xenografts, at ADC doses ranging from 1–10 mg/kg. A dose dependent increase in tubulin occupancy was observed in both xenograft models. At any given dose, the tubulin occupancy for GFP-MCF7 cells was found to be lower than N87 cells, which was comparable to the trend observed in the tumor growth inhibition studies. Of note, since plasma PK of all T-vc-MMAE analytes declined notably at seven days, the extended occupancy of tubulin by MMAE in the tumor is more reflective of the sustained efficacy of ADC compared to any plasma PK. Accordingly, the tubulin occupancy (${\mathrm{Occ}}_{\mathrm{Tub}}$) values were used to build the systems PK-PD relationship.

#### 4.3.4. Linking Intracellular Tubulin Occupancy to Tumor Growth Inhibition

**Figure 6:**
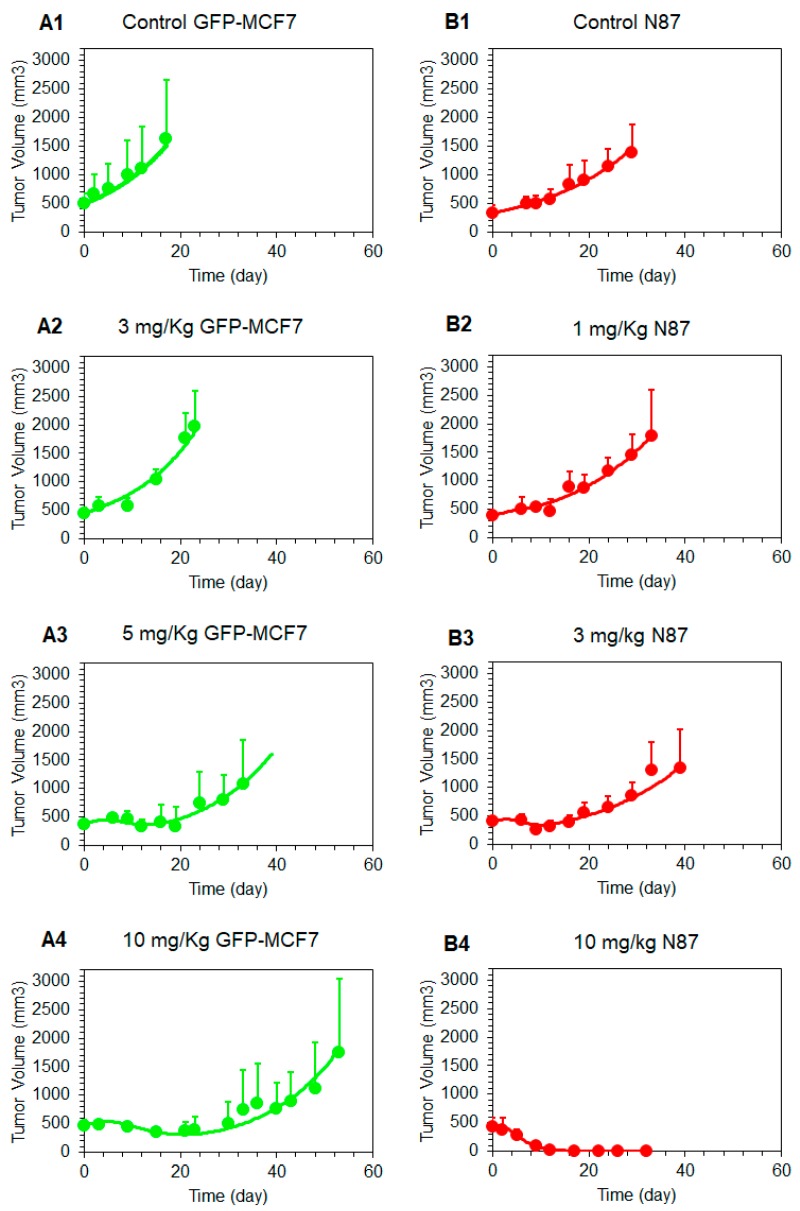
Observed and model predicted tumor growth profiles in GFP-MCF7 (green) and N87 (red) tumor bearing mice in either control group (**A1**, **B1**) or after 1 mg/kg (**B2**), 3 mg/kg (**A2**, **B3**), 5 mg/kg (**A3**), and 10 mg/kg (**A4**, **B4**) T-vc-MMAE.

Figure 6 shows the observed and model fitted tumor growth inhibition profiles generated following the treatment of GFP-MCF7 and N87 xenograft baring mice with various doses of T-vc-MMAE. The observed data was fitted using the complete in vivo systems PK-PD model shown in Figure 1, where all the parameters associated with plasma and tumor PK of ADC were fixed, and only the parameters associated with tubulin occupancy induced tumor cell killing were estimated. The model was able to capture tumor growth inhibition profiles of GFP-MCF7 and N87 xenografts reasonably well and provided robust estimates of PD parameters (Table 1, Figure 6). The model estimated KC50 value was 96.8%, which reflects intracellular occupancy of tubulin required to induce half of the maximum cell killing. This value was very close to the value of 98.3% and 96.1% found for N87 and GFP-MCF7 cells in our previous in-vitro experiments (i.e., $\mathrm{KC}50_{\mathrm{invitro}}^{\mathrm{OCC}}$) [15]. This consistency validates our model-based hypothesis that a very high intracellular occupancy to tubulin by MMAE is required to kill a tumor cell. in the in-vivo PK-PD model estimated value of maximum cell killing rate (Kmax) 1.03 (± 0.1) $\frac{1}{day}$ was also similar to the value of 0.72 and 0.51 $\frac{1}{day}$ estimated for N87 and GFP-MCF7 cells in-vitro (${\mathrm{Kmax}}_{\mathrm{invitro}}^{\mathrm{Cell}}$) [15]. This consistency between in-vitro and in-vivo parameters suggests that one should be able to incorporate the cell-level PK-PD model in the in-vivo setting without significantly altering model structure and parameters.

## 5. Discussion

While ADCs are promising anticancer agents, they are much more challenging to develop compared to antibodies or small molecules due to their complex PK-PD characteristics [26,27]. Despite their tremendous growth, failure rates of ADCs in the clinic remain high, especially for solid tumors. According to a recent statistic, more than 95% of ADCs discontinued from the clinical development in past few years were indicated for solid tumors [5]. Majority of these failures occur in Phase-I due to the lack of efficacy or severe toxicity, which highlights the lack of a robust preclinical-to-clinical translation framework for these molecules. While plasma concentrations of ADCs have been used to drive the efficacy of ADC in the majority of PK-PD relationships published in the literature, ADC exposure in a solid tumor is not in rapid equilibrium with systemic circulation [28,29]. In fact, intratumoral exposures of different ADC analytes could be dramatically different than systemic circulation, suggesting tumor exposure of ADCs should be used to drive the efficacy against solid tumors. Accordingly, we have developed a systems PK-PD model for ADCs that creates a quantitative relationship between plasma and tumor exposures of different ADC analytes and can serve as a robust mathematical tool for successful preclinical-to-clinical translation of ADCs.

In the past we have demonstrated the utility of an ADC PK model to simultaneously characterize plasma and tumor disposition of different ADC analytes, using several ADCs, such as SGN-35 [11], T-DM1 [10,12], A1mcMMAF [13], and Inotuzumab-ozogamicin [30], which differ widely in their linker, payload, and target properties. A detailed sensitivity analysis of this model has revealed that accurate characterization of the disposition of ADC within the cancer cells is essential for a priori predicting the PK of different ADC analytes within the tumor [12]. Consequently, we have recently developed a single-cell systems PK model for ADCs using T-vc-MMAE as a tool compound. Using in vitro experiments, we have validated this model, and have demonstrated that the model is capable of accurately characterizing the relationship between extracellular concentrations of ADC and the concentrations of different ADC analytes within a cell [14]. This cell-level PK model has also been utilized to develop an in-vitro PK-PD model, where intracellular occupancy of the target by the released drug was used to drive the cytotoxicity. With the help of this systems model, it was observed that once the difference in cellular properties (e.g., antigen expression level) were accounted for, the model estimated values for released drug potency and efficacy were very similar between different cell lines [15]. This suggests that accurate characterization of ADC PK parameters leads to more robust estimation of ADC PD parameters.

In this paper, we have expanded the in-vitro PK-PD model toward the in-vivo system. To support the PK-PD model development, mouse xenograft models were developed using GFP-MCF7 and N87 cell lines, and PK and TGI studies were conducted using T-vc-MMAE ADC in these animal models. It was observed that plasma PK profiles for different ADC analytes (i.e., total trastuzumab, unconjugated MMAE, and total MMAE) were identical in GFP-MCF7 and N87 tumor bearing mice. This reflects the minimal contribution of target mediated disposition processes in these animal models at the selected dose of the ADC. In addition, notably faster clearance of the antibody was observed in these animals (Figure 2A1,B1), which could be attributed to Fc-gamma receptor mediated clearance of the antibody in the SCID mice [25]. If desired, this effect can be mitigated by prior treatment with intravenous immunoglobulin (IVIG, ~10 mg) or Fc block (~0.2 mg). Total MMAE profiles in the plasma (Figure 2A1,B1) started with higher concentrations than the antibody, which reflects the initial average DAR value of ~4 in the formulation. However, after ~1-day total MMAE and antibody profiles crossed each other, suggesting average DAR value of the ADC declined below 1 at this time point. The unconjugated MMAE profiles in the plasma (Figure 2A1 and 2B1) were found to be always parallel to the total antibody profiles, validating the formation-rate limited nature of unconjugated drug clearance from the plasma [9].

The tumor exposure of ADC analytes was considerably different than the plasma exposure, and the exposure was also different between the two tumor models (Figure 2). There was a >10-fold difference in the total trastuzumab exposure between the two tumors, which reflects antigen-mediated retention of the antibody in the N87 tumors compared to the GFP-MCF7 tumors. This difference in antibody exposure was however lower than what was observed in the in vitro studies (~ 100-fold) [14] using the same cell lines, which could be due to either altered antigen expression between in vitro and in vivo tumor cells or because of the limited access of the antigen due to heterogeneous distribution of the antibody in the tumor. When unconjugated and total MMAE profiles were compared between the two tumors (Figure 2A2,B2), it was observed that the difference in the exposure was only ~2-fold. A similar effect was also observed during our in vitro studies [14], which is reflective of the saturable intracellular binding capacity of the tumor cells. Despite the higher influx of MMAE molecules inside high antigen expressing cells, only the once bound inside the cell can be retained, and the remaining molecules can quickly diffuse out of the cells. Thus, an enhanced influx of MMAE inside a cell does not necessarily lead to a proportional increase in intracellular exposure of MMAE.

TGI studies revealed that a single dose of 1-10 mg/kg T-vc-MMAE was much more efficacious in N87 tumors compared to GFP-MCF7 tumors. A dose-dependent increase in tumor regression was observed for HER2-high N87 tumors, whereas the effect of ADC was saturated at the higher doses for HER2-low GFP-MCF7 tumors. The observed efficacy of ADC in both the mouse models was considerably prolonged compared to the plasma exposure, and the effect was more synchronous with MMAE exposure in the tumor. This observation underscores the need to use tumor exposure of ADC to develop robust exposure-response relationships that can be successfully translated to the clinic. Our proposed systems PK-PD model is designed to facilitate the development of these relationships.

Figure 1 describes the full in-vivo systems PK-PD model developed by us. The model was able to effectively capture plasma PK of all three analytes of the tool ADC T-vc-MMAE. The model estimated a relatively faster value of MMAE deconjugation rate from the ADC $\left(K_{\mathrm{dec}}^{P}\right)$ [10], suggesting faster decline in the DAR value of T-vc-MMAE over the time. The plasma PK model was extended to have tumor distribution and single-cell disposition of T-vc-MMAE within the tumor. Majority of model parameters were fixed to known values, and only the parameters related to HER2 receptors expression were estimated via model-fitting. The number of HER2 receptors estimated for N87 and GFP-MCF7 cells were found to be 185,000 and 22,400, respectively. While these estimates reflect ~10-fold difference in HER2 expression between the two cancer cells, the in-vivo estimated values were considerably lower than the values measured in-vitro [14,16]. This could be due to several reasons highlighted earlier, like differences in in-vitro and in-vivo systems and limited accessibility of antigen. The tumor PK model was used to estimate intracellular occupancy of tubulin by MMAE, and this parameter was used to develop a novel PK-PD relationship, where single-cell tubulin occupancy governed the killing of tumor cells. To maintain the mass balance, upon the death of each cell the intracellular ADC content was assumed to release outside the tumor cell, and it was allowed to distribute back into another cell or diffuse out of the tumor in the systemic circulation. It was found that the same set of PD parameters was able to capture MMAE induced cytotoxicity of GFP-MCF7 and N87 cells in vivo. Additionally, the estimated single cell potency parameters from in-vivo modeling were very similar to the in-vitro estimates [15]. This observation highlights the need and benefit of developing a systems PK-PD model for ADC, which can account for the changes in the underlying system as the project transitioning from discovery-to-preclinical and preclinical-to-clinical stage of drug development. Exposure-response and dose-response relationships developed based on these models could be more robust and predictive and can facilitate successful clinical translation of ADCs.

In sum, here, we have presented the development of a novel systems PK-PD model for ADCs, which integrates single-cell PK and PD of ADC with a mechanistic tumor distribution model. The presented model has several salient features that differentiate it from previously published models [10,11,13]. The model accounts for a dynamic population of tumor cells and mass-transfer of ADC species among each tumor cell. Additionally, the model also incorporates dilution of intracellular drug content as a function of tumor growth and accounts for the mass-transfer of released drug from dead cells into systemic circulation. Moreover, this model exemplifies the unprecedented utility of using occupancy of the pharmacological target (tubulin) at the site of action to drive the pharmacodynamic effects. Going forward, the presented model could be expanded to account for multiple tumor cell populations, which can help in better characterizing tumor heterogeneity and the bystander effect of ADCs [16]. The presented model is also amicable to incorporation of immune cells, which can help in characterizing the interaction between ADCs, immune-oncology drugs, and the immune cells in the tumor.

## Figures and Tables

**Table 1:** A list of literature derived, or model estimated parameters used for the systems PK-PD model of T-vc-MMAE.

Parameter	Definition	Value (CV %)	Unit	Source
Parameters associated with plasma pharmacokinetics of T-vc-MMAE				
${\mathrm{CL}}_{\mathrm{ADC}}$, ${\mathrm{CLD}}_{\mathrm{ADC}}$	Central and distributional clearances of T-vc-MMAE	0.033 (4.8%), 0.0585 (12.6%)	L/day/Kg	Estimated
$V1_{\mathrm{ADC}}$, $V2_{\mathrm{ADC}}$	Central and peripheral volumes of distribution for T-vc-MMAE	0.084 (7.3%), 0.051 (5.2%)	L/Kg	Estimated
${\mathrm{CL}}_{\mathrm{Drug}}$, ${\mathrm{CLD}}_{\mathrm{Drug}}$	Central and distributional clearances of free MMAE	18.40, 1.84	L/day/Kg	[11]
$V1_{\mathrm{Drug}}$, $V2_{\mathrm{Drug}}$	Central and peripheral volumes of distribution for T-vc-MMAE	0.136, 0.523	L/Kg	[11]
$K_{\mathrm{dec}}^{P}$	Non-specific deconjugation of MMAE from T-vc-MMAE	0.323 (8.8%)	1/day	Estimated
Parameters associated with tumor distribution of T-vc-MMAE				
$R_{\mathrm{Cap}}$	Radius of the tumor blood capillary	8.0	µm	[18,19,20]
$R_{\mathrm{Krogh}}$	An average distance between two capillaries	75.0	µm	[18,19,20]
$P_{\mathrm{ADC}}$, $P_{\mathrm{Drug}}$	The rates of permeability of T-vc-MMAE and MMAE across the blood vessels respectively	334, 21000	µm/day	[18,19,20]
$D_{\mathrm{ADC}}$, $D_{\mathrm{Drug}}$	The rates of diffusion of T-vc-MMAE and MMAE across the blood vessels respectively	0.022, 0.25	cm^2^/day	[18,19,20]
$\varepsilon_{\mathrm{ADC}}$, $\varepsilon_{\mathrm{Drug}}$	Tumor void volume for T-vc-MMAE and MMAE	0.24, 0.44	Unitless	[18,19,20]
$R_{\mathrm{Tumor}}$	Radius of a spherical tumor calculated based on varying tumor volume (TV) where: $\mathrm{TV}\left(t\right)=\frac{4}{3}{*\pi *R}^{3}{}_{\mathrm{Tumor}}$	Dynamic	cm	
Parameters associated with single cell disposition of T-vc-MMAE				
$K_{\mathrm{on}}^{\mathrm{ADC}}$	Second order association rate constant between T-vc-MMAE and HER2 receptor	0.03	1/nM/h	[14]
$K_{\mathrm{off}}^{\mathrm{ADC}}$	First order dissociation rate constant between T-vc-MMAE and HER2 receptor	0.014	1/h	[14]
$K_{\mathrm{int}}^{\mathrm{ADC}}$	Internalization rate of HER2-ADC complex inside the cell	0.11	1/h	[14]
$K_{\deg}^{\mathrm{ADC}}$	Intracellular degradation of T-vc-MMAE in endosomal/lysosomal space	0.353	1/h	[14]
$K_{\mathrm{on}}^{\mathrm{Tub}}$	Second order association rate constant between cytoplasmic MMAE and intracellular tubulin protein	0.0183	1/nM/h	[14]
$K_{\mathrm{off}}^{\mathrm{Tub}}$	First order dissociation rate constant between MMAE-tubulin complex	0.545	1/h	[14]
${\mathrm{Tub}}^{\mathrm{tot}}$	Total concentration of intracellular tubulin in a single cell	65	nM	[11,14]
$K_{\mathrm{in}}^{\mathrm{Drug}}$	First order influx rate of MMAE from extracellular to intracellular space	8.33	1/h	[14]
$K_{\mathrm{out}}^{\mathrm{Drug}}$	First order efflux rate of MMAE from intracellular to extracellular space	0.046	1/h	[11]
${\mathrm{Ag}}_{N87}^{\mathrm{ex}}$, ${\mathrm{Ag}}_{\mathrm{MCF}7}^{\mathrm{ex}}$	Model estimated HER2 receptor count on each tumor cell in N87 and GFP-MCF7 tumors in vivo	185,000 (2.8%), 22,400 (3.2%)	Numbers/Cell	Estimated
Parameters associated with single cell killing of T-vc-MMAE in tumors				
Kmax	First order killing rate of MMAE in each tumor cell (either GFP-MCF7 or N87)	1.03 (31.3%)	1/day	Estimated
KC50	Percentage of intracellular occupancy to tubulin by MMAE which leads to 50% of maximum killing	96.8 (13.2%)	Percentage	Estimated
τ	Transit time associated with the killing	2.03	Day	Estimated
${\mathrm{IIV}}^{\mathrm{Kmax}}$, ${\mathrm{IIV}}^{\tau }$	Inter-subject variability associated with Kmax and ‘Tau’ values assuming log-normal distribution	10.16 (47%), 19.4 (32%)	Percentage	Estimated
γ	Curve-fitting parameter associated with sigmoidal tubulin occupancy-killing relationship	15.02 (38.6%)	Unitless	Estimated
${\mathrm{DT}}^{N87}$, ${\mathrm{DT}}^{\mathrm{MCF}7}$	Doubling time of N87 and GFP-MCF7 tumors	13.5 (11.4%), 10.6 (18.7%)	Day	Estimated

## Supplementary Materials

The following are available online at https://www.mdpi.com/1999-4923/11/2/98/s1, Figure S1. Schematic of the plasma PK model for ADC. Figure S2. Schematics of the tumor distribution model for ADC.
