# Supplementary material for: A Cell-Level Systems PK-PD Model to Characterize In Vivo Efficacy of ADCs
Source: Pharmaceutics. 2019 Feb 25;11(2):98. doi: 10.3390/pharmaceutics11020098 (PMC6409735; doi:10.3390/pharmaceutics11020098)
Supplement: Supplementary file 1 [file pharmaceutics-11-00098-s001.pdf]

pharmaceutics

Supplementary Materials: A Cell-Level Systems PK-PD Model to Characterize In Vivo Efficacy of ADCs

Aman P. Singh, Leiming Guo, Ashwni Verma, Gloria Gao-Li Wong and Dhaval K. Shah

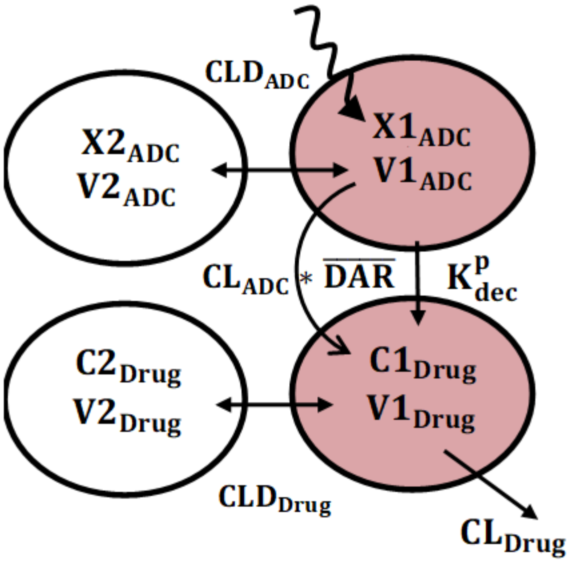

Figure S1. Schematic of the plasma PK model for ADC.

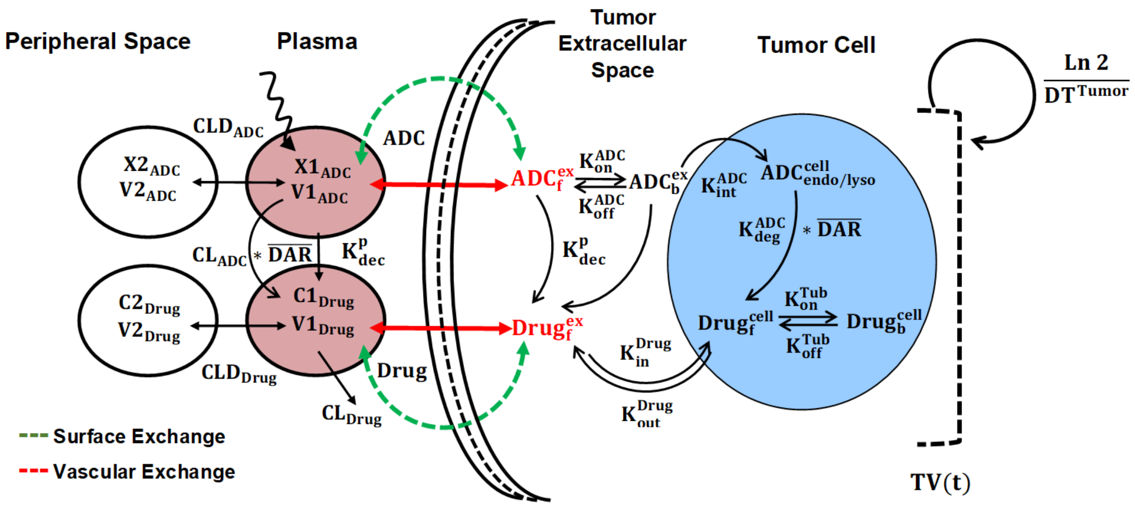

Figure S2. Schematics of the tumor distribution model for ADC.
